# The Evolution of Do-It-Yourself Brain Hacking: From Fringe to Frontier

**DOI:** 10.7759/cureus.85330

**Published:** 2025-06-04

**Authors:** Shaheen E Lakhan

**Affiliations:** 1 Medical Office, Click Therapeutics, Inc., New York, USA; 2 Department of Neurology, Western University of Health Sciences, Pomona, USA; 3 Department of Neurology, A.T. Still University School of Osteopathic Medicine in Arizona, Mesa, USA; 4 Department of Neurology, Morehouse School of Medicine, Atlanta, USA; 5 Department of Bioscience, Global Neuroscience Initiative Foundation, Miami, USA

**Keywords:** biohacking, brain hacking, cognitive optimization, digital therapeutics, microdosing, neuroenhancement, neurotechnology, nootropics, programmable cognition, transcranial stimulation

## Abstract

Do-it-yourself (DIY) brain hacking has evolved from fringe experimentation to a cultural and clinical phenomenon reshaping how individuals interact with their brains. From early self-directed use of racetam nootropics, at-home transcranial direct current stimulation, and meditation apps, to the normalization of microdosing psychedelics, this movement reflects a broader shift toward brain self-optimization. What began as grassroots experimentation now informs regulated interventions. For example, consumer-grade cognitive training tools and neurofeedback devices have inspired FDA-cleared prescription digital therapeutics for conditions such as attention-deficit/hyperactivity disorder, depression, and migraine. Similarly, informal mood tracking and exposure-based journaling practices have evolved into structured cognitive behavioral therapy apps and biosensor-informed just-in-time adaptive interventions. This editorial traces the trajectory of DIY brain hacking across three phases (experimental enthusiasts, consumer biohackers, and clinical convergence) and argues that medicine must shift from skepticism to stewardship. As programmable cognition becomes a clinical reality, and drawing on parallels with other organ systems, guiding this transformation with evidence and ethics is essential to safeguard patient autonomy, efficacy, and safety.

## Editorial

We’re all brain hackers now

We are all brain hackers. The moment you sip your morning coffee to sharpen focus, scroll through a playlist to elevate your mood, or open a meditation app to reduce anxiety, you are engaging in neuroenhancement. These daily choices represent a continuum of self-directed brain modification, attempts to change how we think, feel, or perform using external tools. Table 1 illustrates common brain hacks, such as caffeine [1], music [2], meditation [3], physical exercise [4], and social interaction [5], along with their effects on brain chemistry, neural circuitry, and behavior. What was once a niche pursuit of tech enthusiasts and wellness renegades is becoming a ubiquitous part of modern life.

**Table 1:** Common everyday brain hacks and their neurophysiological and behavioral impacts. This author-generated table describes widely used self-directed behaviors that modulate brain function through well-characterized neurochemical and neural circuit mechanisms, producing measurable changes in cognition, mood, and behavior.

Behavior	Neurochemical impact	Neural circuit impact	Cognitive, mood, and behavioral impact
Caffeine	Adenosine receptor antagonist (A1, A2A), increases dopamine and acetylcholine	Activates prefrontal cortex and basal forebrain networks	Increased alertness, sustained attention, reduced fatigue
Music	Modulates dopamine, endorphins, and oxytocin	Engages auditory-limbic circuits, mesolimbic reward pathway	Mood elevation, memory retrieval, stress reduction
Meditation	Increases GABA, reduces cortisol, enhances serotonin	Regulates default mode and salience networks	Improved emotion regulation, attention, and resilience
Physical exercise	Increases BDNF, dopamine, norepinephrine	Enhances hippocampal neurogenesis and cortical connectivity	Cognitive clarity, improved mood, memory consolidation
Social interaction	Boosts oxytocin, dopamine, serotonin	Activates social cognition and mirror neuron networks	Emotional bonding, stress buffering, cognitive empathy

As a neurologist, neuroscientist, therapeutics developer, and professor, as well as former dean of medicine, I have spent my career at the intersection of brain function, dysfunction, and innovation. I have treated neurodegenerative diseases, explored the neural basis of consciousness, developed novel drugs and digital therapeutics, and educated students and professionals across institutions on how the brain works, structurally and functionally. This lived proximity to the brain has shaped not only how I practice medicine but also how I perceive its future. I have witnessed firsthand how both patients and professionals reach for any means to protect, preserve, and enhance cognitive performance, often outside the traditional medical system.

Today, brain hacking includes everything from nootropic supplements and smartphone-based cognitive training to at-home neurostimulation and experimental psychedelic regimens. While some interventions are evidence-based and clinically validated, others operate in legal or scientific gray zones. Yet, all share a common goal: to reshape brain function in ways that support attention, memory, emotional regulation, creativity, or productivity. The broader cultural context of biohacking has transformed from isolated experimentation to a movement driven by a collective desire to transcend biological limitations. The spread of social media and online communities has only accelerated this shift, normalizing and even glamorizing self-optimization practices.

This editorial examines the evolution of do-it-yourself (DIY) brain hacking from its earliest forms to its present-day intersection with medicine, industry, and ethics. We trace three distinct phases in its development and highlight the challenges and opportunities on the horizon. Most importantly, we argue for a redefinition of the clinician’s role, from passive observer to active guide in this new era of self-directed neuroenhancement. The medical field stands at a critical junction, where ignoring the rising tide of brain hacking could widen the gap between clinical care and consumer behavior. Embracing this phenomenon, with appropriate safeguards, may unlock opportunities for more engaged, personalized, and participatory models of care.

Phase I: experimental enthusiasts (2000s to early 2010s)

The earliest wave of brain hacking grew from the DIY ethos of the Quantified Self movement [6]. Enthusiasts tracked their sleep, mood, heart rate, and cognition with spreadsheets and rudimentary apps. Online forums such as r/Nootropics and DIYbio served as hubs for information exchange and self-experimentation. The early pioneers of this movement were not scientists by training but often had backgrounds in tech, engineering, or health and wellness. Their experiments were driven by curiosity, a dissatisfaction with conventional medicine, and a belief in the plasticity of the brain.

Popular tools in this era included racetams such as piracetam and aniracetam, choline supplements, and early consumer electroencephalography (EEG) devices such as the NeuroSky headset [7,8]. Some individuals built transcranial direct current stimulation (tDCS) rigs at home using 9 V batteries and electrodes, relying on sparse literature and anecdotal guidance. Others adopted regimens of intermittent fasting, cold exposure, or microdosed psychedelics to achieve cognitive “flow states” and improved mental clarity. This period was characterized by a sense of exploration and boundary-pushing. While it lacked scientific rigor, it was foundational in fostering a mindset of neuroplastic experimentation.

Scientific rigor was often lacking. Most interventions were driven by anecdote, biohacker blogs, and trial and error. Nevertheless, this period set the stage for a growing appetite for personal agency in brain optimization. It also foreshadowed ethical and safety challenges that would become more pronounced as the movement matured. There were no established norms, guidelines, or safety protocols. Adverse effects were shared in forums but not systematically tracked. This lack of oversight created a wild west of cognitive enhancement, where results varied wildly and safety was largely assumed rather than proven. From my perspective as a clinical neuroscientist and regulatory strategist, this era foreshadowed the urgent need for guardrails, ethical, scientific, and regulatory, to ensure that curiosity-driven exploration could evolve into safe, scalable innovation.

Phase II: consumer biohacking boom (mid-2010s to early 2020s)

The second phase was marked by commercialization and mainstream adoption. High-profile figures in tech and wellness embraced brain hacking as a pathway to peak performance. Books, podcasts, and branded protocols emerged to support neuroenhancement as a lifestyle. The narrative shifted from underground experimentation to a visible cultural trend. Terms like “biohacker” entered the lexicon of productivity culture, and major media outlets began to profile individuals who used cutting-edge tools to optimize their minds.

Industry responded with a proliferation of consumer neurotechnology and wellness products. Companies released sleek wearable devices promising to boost cognition or relaxation. For example, EEG headbands such as the Muse were marketed to enhance meditation focus by providing real-time brainwave feedback. Cranial electrotherapy stimulators such as Alpha-Stim were promoted to reduce anxiety and improve sleep. Direct-to-consumer tDCS devices, notably the Halo Neuroscience headset, targeted at athletes and learners, claimed to accelerate muscle memory and cognitive training by electrically priming the motor cortex [9]. At the same time, app-based digital tools for mental wellness became household names. Meditation apps (e.g., Headspace, Calm) put guided mindfulness exercises in millions of pockets, supported by studies showing mindfulness can reduce stress and anxiety [10]. “Brain-training” games and cognitive exercise platforms (such as Lumosity and BrainHQ) boomed in popularity, bringing neuroscientific concepts to the smartphone era, though some, such as Lumosity, faced regulatory action over unproven health claims [11]. The global nootropics market also surged, with countless over-the-counter “brain booster” supplements sold as stacks to enhance memory, productivity, and mental energy without crashes.

This era also saw the normalization of microdosing, the sub-perceptual use of psychedelics such as LSD or psilocybin, as a form of creative and emotional optimization [12]. Though largely unsupported by clinical trials at the time, the practice gained traction among professionals seeking a cognitive edge. Self-reported benefits included increased empathy, reduced anxiety, and improved problem-solving skills. Social media platforms became a key vector for spreading microdosing protocols, anecdotal reports, and harm reduction strategies.

While regulation remained limited, the increasing accessibility of brain hacking tools raised red flags. Reports of side effects from tDCS misuse, concerns about long-term neurochemical disruption, and blurred lines between wellness and medical claims began to surface [13]. Regulatory agencies struggled to keep pace with the speed of innovation. The lack of standardization, certification, or clinical validation meant that users were often left to navigate efficacy and safety on their own. Still, the demand for brain-enhancing products and services grew steadily, reinforcing the perception that cognitive optimization was both achievable and desirable.

Phase III: clinical convergence (late 2020s to present)

In recent years, the gap between DIY brain hacking and evidence-based medicine has narrowed. Digital therapeutics and neuromodulatory devices, once fringe concepts, are now entering clinical practice with regulatory oversight. A growing body of peer-reviewed research, coupled with advances in digital health regulation, has enabled the translation of certain biohacking tools into medically sanctioned interventions. 

Notably, the U.S. FDA has cleared a new class of prescription digital therapeutics (PDTs) for neurological and psychiatric conditions, bringing rigor to some interventions that echo earlier DIY approaches [14]. For example, in 2020, the FDA authorized EndeavorRx, a video game-based therapy for children with attention-deficit/hyperactivity disorder (ADHD), the first game-based PDT for any condition. EndeavorRx uses engaging motor and cognitive tasks in an app to improve attention function, a concept that evolved from decades of cognitive training games (and arguably from the brain-training apps popular in Phase II) [15]. By 2024, additional PDTs emerged: Rejoyn, a six-week cognitive-emotional training app for depression, became the first FDA-cleared PDT for major depressive disorder [16]. Another, CT-132, was recently authorized as the first PDT for prevention of episodic migraine, aiming to reduce brain hypersensitivity seen in this condition, after randomized trials showed it significantly reduced migraine days in adults [17]. These platforms incorporate behavioral activation exercises, cognitive training, and adaptive feedback loops, techniques that echo the ethos of biohacking, but now delivered with clinical validation from rigorous trials. By leveraging software delivery and behavioral science, PDTs are beginning to bridge the gap between individualized cognitive strategies and standardized medical treatment.

At the same time, research on neuromodulation techniques such as transcranial electrical stimulation (tDCS, transcranial alternating current stimulation (tACS)), neurofeedback, and closed-loop brain-computer interfaces (BCIs) has gained significant traction [18]. These approaches hold promise not only for treating refractory clinical conditions and restoring lost function but even for enhancing baseline performance in the healthy, blurring the line between therapy and enhancement. In the realm of noninvasive stimulation, for instance, large controlled trials are now evaluating tDCS and tACS for depression, cognitive aging, and other indications, building an evidence base that was absent a decade ago [19]. Neurofeedback training, once a self-experimenter’s hobby, is being refined as a potential therapy in ADHD, anxiety, and even optimal performance programs, with studies showing it can induce beneficial neural plasticity under certain conditions. Meanwhile, companies such as Neuralink and Synchron are pushing the frontier of implanted BCIs, envisioning systems to restore communication to paralyzed patients, enhance memory, or allow direct control of external devices by thought [20,21]. In 2023, Neuralink received FDA authorization to begin human trials of its high-bandwidth implantable BCI, a milestone suggesting that ultra-hardware-intensive “brain hacking” may soon enter clinical evaluation [20]. Synchron’s stentrode device, which is implanted via blood vessels rather than open brain surgery, has already been tested in human patients with amyotrophic lateral sclerosis to enable texting and device control by neural signals, with encouraging early results [21]. As these neurotechnology tools advance, the very notion of what constitutes “treatment” versus “enhancement” will continue to blur. The idea of restoring function is being reimagined as upgrading function, with neural implants and adaptive stimulation protocols pushing the boundaries of what medicine can deliver for cognition and performance.

Regulatory bodies are slowly evolving in response. The FDA and other agencies have started to define clear pathways for evaluating software as a medical device, as in PDTs, including software that influences mental or cognitive states [14]. In the United States, regulatory history has been made with frameworks for differentiating PDTs and wellness apps, ensuring they meet safety and efficacy standards akin to drugs and devices. The European Union (EU), through guidance documents (such as MDCG 2019-11), similarly clarified how health apps and artificial intelligence (AI)-driven software can qualify as medical devices under EU regulations [22]. Ethics committees and neurotechnology advisory boards are also being established to address novel concerns around autonomy, informed consent, privacy, and the long-term impact of cognitive enhancement tech. Leading neuroscientists and ethicists have called for formal “neuroethical” guidelines to protect fundamental human rights such as mental privacy and agency in the face of BCIs and AI that interact with the brain [23]. Clinical trial designs for invasive neurotech now routinely include independent ethical oversight to monitor for unintended personality or mood changes. Moreover, the integration of patient-reported outcomes, biometric monitoring, and real-world data into regulatory decisions is shaping a new framework for brain-focused therapeutics, emphasizing continuous post-market evaluation of how these interventions perform in everyday life.

The brain is not alone: precedents in other organ systems

To better understand the trajectory of brain hacking, it is helpful to examine how other organ systems have followed similar arcs, from fringe experimentation to clinically accepted, and even routine, interventions. History shows that medicine has repeatedly incorporated practices that began as outsider-driven innovations or grassroots experiments. These precedents validate the current moment in neuroenhancement and provide a roadmap for its clinical integration.

In endocrinology and metabolic health, crude hormonal manipulation, extreme diets, and biometric self-tracking were early hallmarks of DIY experimentation. These grassroots practices anticipated the development of closed-loop insulin pumps, continuous glucose monitors, and structured diabetes reversal programs that now form the foundation of precision metabolic care [24,25]. In cardiology, techniques such as heart rate variability training and breath-based autonomic regulation predated the emergence of smartwatches capable of detecting arrhythmias, AI-driven rhythm management platforms, and baroreflex activation therapy for resistant hypertension [26,27]. What was once intuitive self-regulation has become algorithm-guided cardiovascular optimization. In pulmonology, practices such as yoga-based breathwork and even didgeridoo playing for airway tone enhancement have influenced validated therapies such as pulmonary rehabilitation, adaptive ventilatory support, and hypoglossal nerve stimulation for obstructive sleep apnea [28,29]. In gastroenterology, unregulated probiotic use, elimination diets, and at-home fecal microbiota transplants anticipated today’s structured dietary protocols (e.g., low-FODMAP), prescription probiotics, and FDA-approved fecal microbiota therapies [30,31]. Increasingly, neuromodulation of the gut-brain axis is also being clinically explored for functional bowel disorders [32]. In reproductive medicine, informal fertility tracking using basal body temperature and hormone self-monitoring set the stage for FDA-cleared contraceptive apps and AI-assisted in vitro fertilization protocols that tailor treatment with remarkable precision [33]. Finally, psychiatry has seen once-personal coping strategies, mood logs, exposure therapy, journaling, inform the development of PDTs, cognitive-behavioral therapy apps, biosensor-informed just-in-time adaptive interventions, and immunopsychiatry approaches that target systemic inflammation in mental illness [34,35]. Table 2 illustrates this progression, showing how self-directed practices have translated into established clinical counterparts across these organ systems.

**Table 2:** Translational pathways from self-directed interventions to evidence-based clinical practices across organ systems. This author-generated table summarizes how grassroots, DIY-style interventions aimed at optimizing specific organ systems, originally developed outside traditional medicine, have influenced and often been adopted into mainstream clinical practice. Each organ system shows a trajectory from speculative self-experimentation to validated, regulated therapeutic applications. Many of these clinical tools now reflect scientific refinement of what were once viewed as fringe or pseudoscientific techniques.

Organ system	Early self-directed interventions	Established clinical counterparts
Endocrine and Metabolic	Unregulated hormone use, intermittent fasting, ketogenic diets, thyroid enhancers	Closed-loop insulin pump systems, structured diabetes reversal programs, hormone replacement therapies
Cardiology	Heart rate training, heart rate variability biofeedback, breath regulation	Wearable arrhythmia detection, artificial intelligence (AI)-guided rhythm management, baroreflex activation therapy
Pulmonology	Breathwork (e.g., pranayama), didgeridoo therapy, vagus nerve self-stimulation	Continuous positive airway pressure devices, adaptive ventilators, hypoglossal nerve stimulation for sleep apnea
Gastroenterology	Elimination diets, unregulated probiotics, unsupervised fecal transplants	Low-fermentable oligosaccharides, disaccharides, monosaccharides, and polyols diet, prescription probiotics, U.S. FDA-approved fecal microbiota transplantation, vagus nerve modulation
Reproductive Medicine	Calendar-based fertility tracking, basal temperature charting, cycle journaling	FDA-cleared digital contraceptive applications (e.g., Natural Cycles), home hormone monitoring, AI-assisted in vitro fertilization
Psychiatry	Mood tracking, journaling, exposure practices	Prescription digital therapeutics; e.g., Rejoyn), cognitive-behavioral therapy apps, biosensor-informed just-in-time adaptive interventions

These precedents demonstrate that innovation often begins at the margins. As with other domains, the frontier of brain enhancement is already influencing the future of care, and its responsible clinical translation is both plausible and imperative.

Conclusion: stewardship over skepticism

The trajectory of brain hacking, from DIY experimentation to clinical adoption and now visionary societal integration, demands a new posture from medicine, science, and policy. What was once dismissed as fringe behavior is undeniably influencing the future of cognitive care, mental health, and human performance. We must recognize that cognitive self-optimization is not just a cultural trend but part of a foundational shift in how people engage with their own brains.

This progression echoes the path of other organ systems once deemed “unhackable.” In endocrinology, hormone manipulation began with crude animal extracts and unregulated supplementation, yet evolved into precision therapies such as closed-loop insulin pumps and continuous glucose monitors, now staples of modern diabetic care. Cardiology witnessed a similar transformation, as early personal tracking and biofeedback experiments presaged today’s smart pacemakers, wearable arrhythmia detectors, and algorithm-guided hypertension management. Pulmonology, gastroenterology, psychiatry, and reproductive medicine have all followed this pattern: outsider-driven interventions matured into life-changing standards of care. The nervous system, long protected by a mystique of inaccessibility, is now undergoing the same evolution.

Clinicians must move beyond a stance of skepticism or passive tolerance. As patients increasingly arrive with printouts of their smartwatch EKGs, logs of at-home tDCS sessions, microdosing schedules, or wearable-derived attention graphs, healthcare professionals need to be equipped to respond with both scientific insight and compassionate guidance. Dismissing or disparaging patients’ self-experiments will only drive a deeper wedge between the medical establishment and an empowered public. Instead, clinicians should strive to become stewards of safe and effective brain enhancement, helping individuals discern evidence-based approaches from snake oil, monitoring for adverse effects, and integrating beneficial “home hacks” into holistic care plans where appropriate.

Regulatory frameworks, too, must evolve, not only to protect users from harm but to proactively enable access to validated tools. This means developing agile pathways to evaluate direct-to-consumer neurotechnologies and supplements, crafting guidelines for ethical use of cognitive enhancers, and updating training for providers to address patient use of such tools. Research must expand beyond traditional pathology to investigate enhancement, resilience, and flourishing. In addition to treating disease, we should ask: How can we help healthy brains work even better or stay at peak longer? The line between treatment and enhancement will keep shifting, and it is incumbent on the scientific community to study both sides of that line rigorously.

The next decade will not merely be about regulating or legitimizing brain hacking, it will be about designing the mental infrastructure of a cognitively empowered society. The rise of programmable cognition, closed-loop mental health systems, and neuroadaptive environments will challenge us to redefine what it means to be “healthy,” “capable,” and even “human.” If medicine is to remain relevant in this coming era, it must adopt a stewardship mindset: not aiming to control this movement from above but to shape it collaboratively for the collective good. We have an opportunity to channel the energy of the brain hacking movement into evidence-based, equitable innovations that could benefit many, but only if we engage with it openly and creatively.

As someone working daily at the frontier of digital brain medicine, I believe clinicians and scientists must adopt a proactive role in guiding this movement, not merely watching from the sidelines but co-designing its future. Ultimately, the brain is no longer a black box or a sacred frontier. It is the next programmable system: one we must approach not with fear or hype, but with precision, humility, and shared purpose. The question is not whether we will enhance the brain, but whether we will do so wisely. By learning from the past and embracing a role as stewards, we can help ensure this new frontier of human enhancement unfolds responsibly, safely, and in service of our highest potential.
